# Epidemiological study of tick infestation in buffalo of various regions of district Khairpur, Pakistan

**DOI:** 10.14202/vetworld.2017.688-694

**Published:** 2017-06-24

**Authors:** Farzana Abbasi, Imtiaz Hussain Raja Abbasi, Tahmeena Fakhur Nissa, Zohaib Ahmed Bhutto, Muhammed Asif Arain, Rab Nawaz Soomro, Farman Ali Siyal, Sarfraz Ali Fazlani

**Affiliations:** 1 Department of Zoology, Faculty of Natural Sciences, Shah Abdul Latif University, Khairpur, Pakistan; 2 Department of Animal Nutrition, College of Animal Science and Technology, Northwest A & F University, Yangling, 712100, China; 3 Department of Chemistry, Shah Abdul Latif University, Khairpur, Pakistan; 4 Department of Animal Husbandry, Faculty of Veterinary and Animal Sciences, Lasbela University of Agriculture, Water and Marine Sciences, Uthal, 3800, Pakistan; 5 Department of Animal Nutrition, Faculty of Animal Husbandry and Veterinary Sciences, Sindh Agriculture University, Tandojam, Pakistan

**Keywords:** *Argasidae*, buffalo, identification, *Ixodidae*, population, prevalence, ticks, tick infestation

## Abstract

**Aim::**

The aim of this study was to determine the epidemiological infestation and identification of *Ixodidae* and *Argasidae* ticks species in buffalo of different parts of district Khairpur, Pakistan.

**Materials and Methods::**

A total of 720 Water buffaloes from three tehsils (subdivisions) were selected randomly and examined from organized and unorganized dairy farms for tick infestation in district Khairpur, Pakistan. This epidemiological survey was conducted during April to September 2015.

**Results::**

The overall mean population and preferred site of tick attachment to infested animals, in Gambat, Sobhodero, and Kot Diji tehsils, were observed on different body parts. The primary body area of infestation by ticks (head, thorax, abdomen, udder, and tail) ranged from highest in tail and udder part compared to lowest in the abdomen, head, and thorax. In all study areas, the infestation was higher (p<0.05) in tail and udder than other parts of the body. In all the study areas, the overall highest population was found in the month of July. In addition, we first time identify four new species of ticks (*Hyalomma anatolicum, H. anatolicum excavatum, Hyalomma Ixodes excavatum*, and *Ixodes ricinus*) in district Khairpur, Pakistan.

**Conclusion::**

Results of this study provide additional information of epidemiological tick infestation, and will be helpful for evolving effective control policy for the management of tick infestation in study district.

## Introduction

Buffalo (*Bubalus bubalis*) primitively domesticated in Asia for milk and meat purpose. The phylogenetic records described that the buffalo originated approximately 4000-5000 years ago from China to India [1]. The estimated population of domesticated buffalo in Pakistan about 23.4 million, and three breeds (Kundi, Nili-Ravi, and Azi-Kheli) of water buffaloes were commonly found [2]. Pakistan produces about 1.6 billion tons of milk and 4 million hides per year from buffaloes [3]. A number of environmental factors such as diet, feeding regime, housing, climate, season, heat stress, parasitic burden, and disease status affect milk production of dairy buffalo. Among many constraints, parasitism is thought to be a major hindrance in the development of livestock population including buffaloes. In Pakistan during this decade parasitism is burning issue which causes health problems for domestic animals production, based on climate, water availability, land use, and physiographic parts of Pakistan offer favorable environmental conditions for ticks, which can infest a variety of hosts and transmit diseases to humans, livestock, and companion animals [4]. In comparison with other seasons, ticks are found actively during summer and spring [5]. There are diverse ticks, and tick-borne diseases occur in domestic animals transmitted by ticks in Pakistan [6,7]. Tick and tick-borne diseases are worldwide problems for livestock health, and its severity depends on the area, host population, species involved, socioeconomics and adaptation of advanced technologies for control measures [7-9]. Losses occur due to ticks either directly through tick burden, loss of blood, damage to hide and udder and infection due to toxins, or indirectly though the mortality or weakness cause by the disease transferred by, or associated with the ticks. The annual global economic losses due to tick infestation have been expected US$14000-18000 million, and the price of management of TTBDs in livestock of Pakistan and India is as high as US$ 498.7 million per annum [10]. The most common combined effects of tick and tick-borne infection in Pakistan dairy industry is the drop in milk yield and the quality of the hides and leather industry.

The tick infestation is the most common and considered to be economically important in a domestic animal, and reported studies conducted in different species such as ruminants [11], sheep [12-15], goat [12,15,16], dairy cattle [15-18], and buffalo [16-19]. In our country, there were limited research reports concerning the prevalence of tick infestation found in domestic animals particularly in ruminants, and those studies were conducted in few agro-climatic regions [20-27]. A recent study conducted in two districts of lower Punjab indicates that the prevalence of bovine tick infestation (BTI) exceeds 50% [28,29]. The diverse agro-climatic conditions, animal husbandry practices, and pasture management largely determine the variability and severity of prevalence of BTI [30] which necessitates the need for an epidemiological survey of different agro-climatic zones of the country which may provide better understanding and strategic control to the small holder dairy farming community of Pakistan.

As for as we can ascertain no report is available on the prevalent tick species causing BTI in district Khairpur, upper Sindh Pakistan.

Therefore, keeping in view the importance of the subject this study was conducted with the objective of exploring the epidemiological infestation of the tick in buffalo at three tehsils of district Khairpur, Pakistan. Furthermore, this study provides additional information for the development and modulation control and curative measures, resulting in increased production and economic stability.

## Materials and Methods

### Ethical approval

The current study was carried out in strict accordance with the recommendation of the institutional ethical committee (Faculty of Natural Sciences, Shah Abdul Latif University, Khairpur, Sindh, Pakistan). All procedures and experiments compiled with the guideline and were approved by the institutional committee with respect to the animal experimentation and care of animals under study, and all standard procedures were followed.

### Location and climate of study area

**Figure-1:**
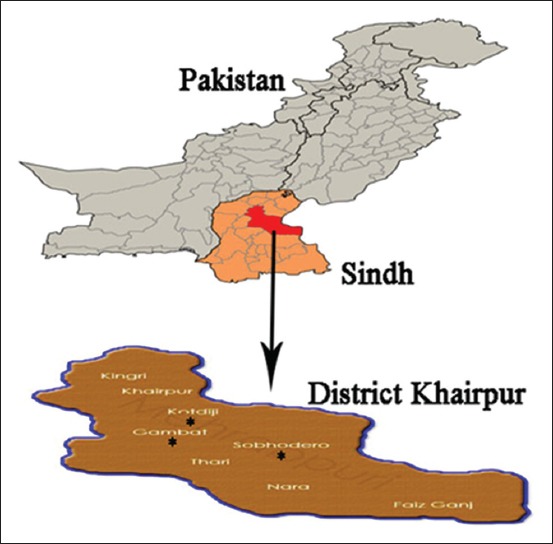
Map of Pakistan, Sindh Province and district Khairpur. *Pointed out the study areas (subdivisions).

**Figure-2:**
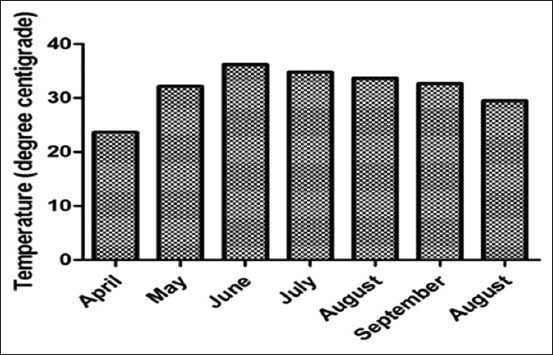
The monthly average of temperature in study district.

The study was conducted at district Khairpur, Sindh Province (Pakistan). The Khairpur district is situated at the south of the Indus River in the Sindh province, and eastern part consists of the Nara Desert. It is extended from the latitudes 27.53°N to 68.77°E longitudes. It covers the geographical area of 15, 910 km^2^ and lies between altitudes 50 m above sea level. District Khairpur has eight subdivisions (Tehsils), and this study focuses on the three subdivisions of study district which includes Gambat, Sobhodero, and Kot Diji (Figure-1). This cross-sectional epidemiological survey was conducted during the summer season from April to September 2015. The average monthly temperature of study district during the given study period mentioned in Figure-2. The climate of the study area is extensively hot and dry in summer, temperature ranges in between 27°C and 47°C, and the estimated average annual rainfall in the area is 12 mm.

### Specimen collections

For ticks collection and for tick infestation 720 water buffaloes were examined, randomly about (n=20) buffaloes from both organized and unorganized farm were selected for sample collection at every farm, with 5-10 year age groups. The collection was carried out fortnightly interval basis in morning and evening time. The ticks were searched by passing hand throughout on the animal coat, and the different stages of ticks (larva, nymph, and adult) were collected with the help of forceps and brush without damage their mouthpart. Ticks were collected from different body parts of buffaloes: Head, thorax, abdomen, udder, and tail regions. These areas were selected due to the high density of tick attachment. The collected ticks were transferred into a plastic bottle containing 70% ethyl alcohol and 30% glycerin for preservation and brought to the laboratory for further studies. Required information such as date of collections, place of collection, and body site of collection, species, and breed of the host was recorded.

### Identification of tick species

Morphological study for species identification of ticks was conducted with the help of high power microscope in the laboratory of the Department of Zoology, Faculty of Natural Science, Shah Abdul Latif University Khairpur. Identification up to species level was made following identification keys and checklists previously described [31,32]. Prevalence (P) was quantified using the equation: P = No. of infested cases during specified period/population at risk during that specified time period × 100.

### Statistical analysis

The data obtained from the prevalence of tick infestation were analyzed by using statistical software, and data presented as mean and standard deviation. Significant level was considered at p<0.05 level between various groups.

## Results

### Tick prevalence at tehsil Gambat

**Figure-3:**
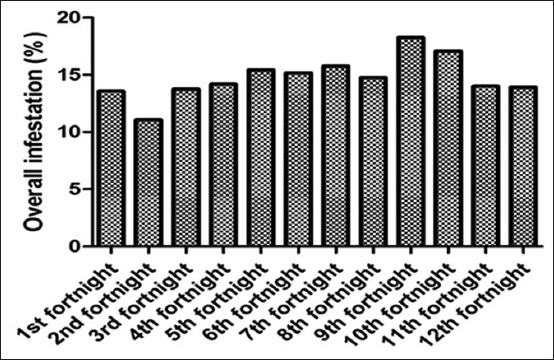
Overall tick infestation in tehsil Gambat.

Among the buffalo population inspected a significantly highest (p<0.05) infestation rate were observed in September (2.19±0.84) and least in May (1.33±0.39) (Figure-3). However, the overall mean population and preferred site of tick attachment to infested animals, at tehsil Gambat, were observed on the head region (0.55±0.29), thorax (0.41±0.25), abdomen (0.53±0.29), udder (3.48±0.73), and tail (3.92±0.71). The analysis of variance showed the significant difference (p<0.001) among all body parts of ticks infestation were recorded, while the nonsignificant difference (p<0.001) was observed among the dates, throughout the summer season (Table-1).

**Table-1:** Tick infestation in different body parts (Tehsil Gambat).

Dates	Head	Thorax	Abdomen	Udder	Tail
20-04-15	1.10±0.43	0.15±0.11	0.85±0.49	2.65±0.42	3.40±0.43
05-05-15	0.35±0.18	0.20±0.12	0.40±0.20	2.40±0.67	3.65±0.79
20-05-15	0.60±0.34	0.40±0.23	0.40±0.27	3.20±0.68	3.15±0.59
05-06-15	0.65±0.35	0.35±0.24	0.50±0.26	3.40±0.74	3.75±0.62
20-06-15	0.50±0.28	0.60±0.34	0.65±0.36	3.80±0.72	3.55±0.74
05-07-15	0.45±0.26	0.55±0.32	0.40±0.20	3.40±0.70	3.95±0.57
20-07-15	0.50±0.28	0.60±0.32	0.70±0.38	4.65±0.83	3.70±0.76
05-08-15	0.80±0.43	0.25±0.I8	0.55±0.32	3.30±0.75	3.60±0.83
20-08-15	0.40±0.24	0.40±0.27	0.50±0.25	3.95±0.85	6.10±0.61
05-09-15	0.40±0.27	0.50±0.31	0.55±0.27	3.55±0.77	5.45±0.81
20-09-15	0.35±0.22	0.40±0.28	0.40±0.24	3.25±0.81	4.65±0.86
05-10-15	0.45±0.22	0.50±0.28	0.45±0.24	4.20±0.81	2.05±0.86
Mean and SE	0.55±0.29^b^	0.41±0.25^b^	0.53±0.29^b^	3.48±0.73^a^	3.92±0.71^a^

### Tick prevalence at tehsil Sobhodero

**Figure-4:**
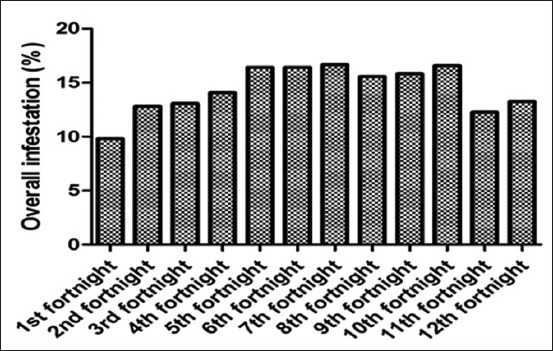
Overall tick infestation in tehsil Sobhodero.

Among the all animal examined in this study, the data showed (Table-2) that the overall mean of tick infestation rate in different region of the body such as head region (0.42±0.27), thorax (0.35±0.22), abdomen (0.50±0.26), udder (3.83±0.71), and tail (3.59±0.74), however, the highest population was found in July (2.00±0.79), and the lowest was recorded in April (1.18±0.78) (Figure-4), which found to be non significantly (p<0.999) variation. The analysis of variance showed the significant difference among all body parts effected with the ticks (p<0.001, <0.05).

**Table-2:** Tick infestation in different body parts (Tehsil Sobhodero).

Dates	Head	Thorax	Abdomen	Udder	Tail
25-04-15	0.50±0.33	0.35±0.22	1.20±0.34	1.95±0.39	1.85±0.53
10-05-15	0.55±0.31	0.30±0.21	0.80±0.26	2.75±0.51	3.30±0.66
25-05-15	0.50±0.29	0.40±0.23	0.60±0.35	3.30±0.63	3.05±0.60
10-06-15	0.40±0.27	0.35±0.24	0.35±0.22	3.65±0.72	3.70±0.59
25-06-15	0.40±0.24	0.40±0.24	0.45±0.29	4.35±0.66	4.25±0.82
10-07-15	0.45±0.22	0.55±0.31	0.30±0.18	5.30±0.76	3.25±0.79
25-07-15	0.40±0.24	0.45±0.29	0.40±0.24	4.80±0.84	3.95±0.86
10-08-15	0.35±0.24	0.45±0.29	0.30±0.18	3.95±0.90	4.30±0.93
25-08-15	0.35±0.26	0.30±0.18	0.40±0.28	4.35±0.84	4.10±0.77
10-09-15	0.45±0.29	0.25±0.18	0.40±0.24	4.95±0.73	3.90±0.86
25-09-15	0.35±0.26	0.20±0.16	0.35±0.24	2.75±0.67	3.70±0.80
10-10-15	0.30±0.22	0.20±0.16	0.40±0.28	3.85±0.87	3.70±0.17
Mean and SE	0.42±0.27^b^	0.35±0.22^b^	0.50±0.26^b^	3.83±0.71^a^	3.59±0.74^a^

### Tick prevalence at tehsil Kot Diji

**Figure-5:**
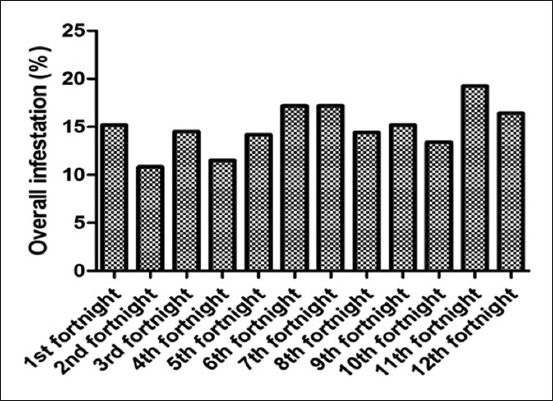
Overall tick infestation in tehsil Kot Diji.

Significant differences (p<0.001) were recorded in the all body parts (p<0.0.05) of tick infestation (Table-3). The overall mean populations were observed on the head region (0.48±1.15), thorax (0.40±1.14), abdomen (0.52±1.29), udder (4.01±3.43), and tail (3.55±3.31). The highest monthly prevalence was found in July (2.31±0.50) while the least were recorded in May (1.30±0.43) (Figure-5) which showed that non significantly different. The analysis of variance showed the nonsignificant difference (p<0.998) throughout the summer season (p<0.05).

**Table-3:** Tick infestation in different body parts (Tehsil Kot Diji).

Dates	Head	Thorax	Abdomen	Udder	Tail
30-04-15	1.30±0.45	0.45±0.23	1.15±0.53	2.85±0.52	3.35±0.53
15-05-15	0.55±0.27	0.45±0.31	0.60±0.30	2.65±0.69	2.25±0.56
30-05-15	0.55±0.29	0.55±0.29	0.65±0.33	3.75±0.65	3.20±0.58
15-06-15	0.40±0.24	0.35±0.22	0.30±0.18	2.90±0.66	2.95±0.59
30-06-15	0.40±0.27	0.35±0.24	0.40±0.24	3.60±0.77	3.75±0.82
15-07-15	0.50±0.26	0.45±0.26	0.65±0.33	4.40±0.84	4.30±0.70
30-07-15	0.45±0.29	0.45±0.29	0.30±0.18	4.75±0.87	4.35±0.91
15-08-15	0.60±0.34	0.30±0.18	0.35±0.26	4.60±0.84	2.80±0.77
30-08-15	0.20±0.16	0.35±0.26	0.45±0.26	4.15±0.83	3.95±0.78
15-09-15	0.25±0.18	0.25±0.18	0.40±0.27	4.45±0.88	2.70±0.91
30-09-15	0.25±0.18	0.40±0.28	0.50±0.29	6.10±0.79	4.30±0.96
15-10-15	0.25±0.18	0.45±0.31	0.45±0.29	3.95±0.88	4.75±0.78
Mean and SE	0.48±1.15^b^	0.40±1.14^b^	0.52±1.29^b^	4.01±3.43^a^	3.55±3.31

### Taxo-morphological identification of tick species at district khairpur

#### Hyalomma anatolicum

**Figure-6:**
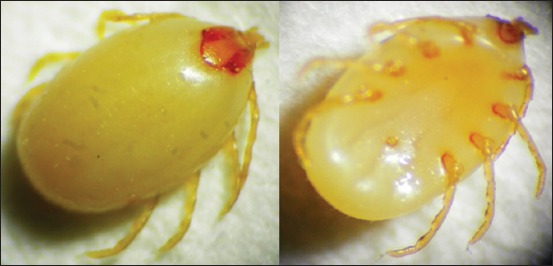
Morphological characteristics of *Hyalomma anatolicum* tick. Female, dorsal side (left) and ventral side (right).

Adult *H. anatolicum* Legs are pale/whitish in color, female scutum is dark brown in colored with smooth at the posterior margin and the mouth part long and forward. The legs are pale whitish in color compare with scutum, the genital aperture is anterior. Festoons and palps are also found (Figure-6).

#### H. anatolicum excavatum

**Figure-7:**
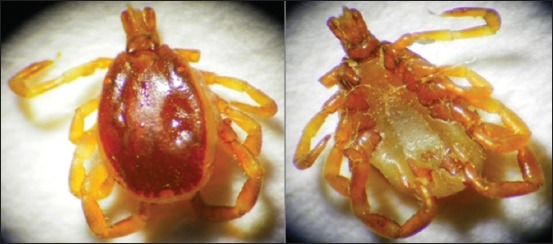
Morphological characteristics of *Hyalomma anatolicum excavatum* tick. Male dorsal view (left) and ventral view (right).

The adult male body of *H. anatolicum excavatum* contains scons cutum, legs are pale white in color, eyes and palps are present. Festoons are pale white in color, and mouth parts are forwarded (Figure-7).

#### Hyalomma Ixodes excavatum

**Figure-8:**
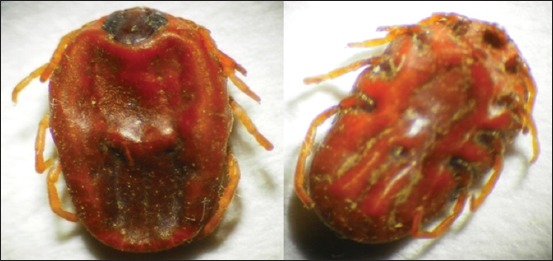
Morphological characteristics of *Hyalomma Ixodes excavatum* tick. Female dorsal side (left) and ventral side (right).

Adult *H. Ixodes excavatum* legs are white in color, female scutum is dark brown in colored and mouthparts are short and downward. The genital aperture is present between first and second coxae (Figure-8).

#### Ixodes ricinus

**Figure-9:**
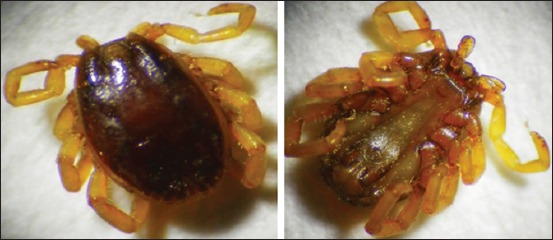
Morphological characteristics of *Ixodes ricinus* ticks. Male dorsal side (left) and ventral side (right).

The adult *I. ricinus* male body contains scons cutum having short and downward mouth parts. Scapular grooves are present; the anal groove is long and posterior in position. Eyes and palps are also present (Figure-9).

## Discussion

The prevalence of tick infestation is a worldwide problem also found in Pakistan. The hot and humid conditions excluding winter were suitable for wide variety of blood sucking parasites especially for ticks [4]. A recent study conducted in two districts of lower Punjab indicates that the prevalence of BTI exceeds 50% [28,29]. In our study, the prevalence rate was lower than the forehead reports. The variation and severity in prevalence of BTI in a specific geographical area possibly depends on the diverse agro-climatic conditions, animal husbandry practices, population of the animals (dense or free), species involved, agents, pasture management, host population, socioeconomic and whether advance technologies for control measures practiced or not [30]. The relationship between host and ticks was depending on season and time for the growth and development of ticks [33]. Among all animal examined in this study, however, the highest population was found in July, and the lowest was recorded in April which is according to the previous reports in our ­country [4,25,27,30], who found prevalence rate of ticks in June to August months. One report from another country also stated that tick infestation was high in summer as compared to winter [34]. The summer temperature of Pakistan in general and Sindh province specifically (38-48°C) is suitable for the activity, growth, development, and reproduction of ticks. In our study, the lower prevalence rate was seen. The lower host susceptibility found in buffalo as compared to previously documented reports in cattle [11] and in ruminants [35], might be due to thicker skin. The buffalo has swampy habitat, and because of wallowing most of the ticks dropped. That’s why dewlap, tail and to soften areas were compared with the whole body. We also investigated the ticks in soften tissue of the body likewise other reported research, the ticks were found on different body parts of buffaloes such as ear, neck, tail, and udder [36]. The 850 species of ticks divided into *Ixodidae* (hard ticks) and Argasidae (soft ticks) families, most commonly found in domestic animals. The *Ixodidae* family consists of 700 while *Argasidae* family consists of 170 species [37]. In the study area, we identified *H. anatolicum*, *H. anatolicum excavatum, Hyalomma Ixodes excavatum*, and *I. ricinus* species of ticks. The most widespread tick species in our country are *Boophilus, Rhipicephalus, Haemaphysalis*, and *Hyalomma*. The previously reported tick species from a different area of our country are *Haemaphysalis*, *Rhipicephalus*, *Boophilus*, *H. anatolicum, Hyalomma marginatum*, and *Rhipicephalus microplus* [4,25,27,29]. Some species of ticks in the study area were different as compared to other species discovered in various areas of our country. The distribution of various species of ticks in different areas possibly depends on the diverse agro-climatic conditions and different species of domestic animals reared in those areas. In general, most of the ticks in this study infested sites with shorter hair and thinner skin (abdomen specially dewlap and tail). These sites promote penetration of tick mouth parts and facilitate better access to the circulation for feeding. It can be concluded that tick infestation is a predominant problem of buffalo population of district Khairpur, Pakistan.

## Conclusion and Recommendation

It was concluded that buffalo ticks population was observed highest, infestation frequency of tick contrast according to animal farms and their congested population, animal performance was noted on the basis of milk production, growth and score ratio decrease day by day. It was detected during the inspection at different sites the overall mean population of tick was observed significantly different from one place to another. Moreover, during the experiment, we identify new tick species; *H. anatolicum; H. anatolicum excavatum; H. Ixodes excavatum; I. ricinus*, on buffalo. The study showed that external parasite infestation adversely affects the health status and pay to decrease milk and meat production of infested animals. Furthermore, it was observed that the farms in the good hygienic condition the infestation rate are less compare with the poor hygienic condition. To effective control of tick infestations, *Acaricide* treatment should be suggested twice in rainy period and once each in summer and winter seasons. The treatment of animal sheds and good hygienic practices including good management and the burning of floors of animal sheds for effective control should be recommended.

## Authors’ Contributions

FA: Conceived the idea and conducted the research. TFN and IHRA: Helped during sample collection and laboratory work. RNS and SAF: Helped to find out the literature and perform statistical analysis. MAA and ZAB: Wrote the manuscript. FAS critically revise the final manuscript. All authors read and approve the final draft for publication.
